# Supplementary material for: A robot intervention for adults with ADHD and insomnia–A mixed-method proof-of-concept study
Source: PLoS One. 2023 Sep 1;18(9):e0290984. doi: 10.1371/journal.pone.0290984 (PMC10473504; doi:10.1371/journal.pone.0290984)
Supplement: S1 File — (DOCX) [file pone.0290984.s002.docx]

Interview guide – the experience

We are interested in your experience of the intervention.

1. Please describe your sleep problems before you tested the sleep robot?
2. Please describe a typical night before the sleep robot?
3. Please describe your current sleep pattern after the sleep robot?
4. If you think back on the first night with the sleep robot, please tell me what happened?
5. How have you used the sleep robot? (Where? For how long? In what position?) Did your way of using it change over time?
6. Please continue to describe your experiences during the period with the sleep robot?
7. The experiences that you describe, have they affected your everyday life in any way?
8. Please describe your thoughts about your time with the sleep robot?
9. What was your expectations? Was it like you anticipated?
10. Did your sleep problems change during the period with the sleep robot? (What do you think caused the change?)
11. If you improved… was it in terms of insomnia symptoms, symptoms of adhd, anxiety or depression or something else? What improvement came first?
12. If the intervention was helpful, why do you think it was helpful?
13. Have you ever tested any other treatments for your sleep problems?
14. Did you experience any negative effects of the sleep robot? Was there something that did not work?
15. Was it difficult to use the sleep robot in any way?
16. Do you do anything differently now after the period with the sleep robot, that you did not do before?
17. Have you had any effects of the sleep robot, good or bad, that we haven’t talked about? Anything else we should know about?
