# Supplementary material for: A robot intervention for adults with ADHD and insomnia–A mixed-method proof-of-concept study
Source: PLoS One. 2023 Sep 1;18(9):e0290984. doi: 10.1371/journal.pone.0290984 (PMC10473504; doi:10.1371/journal.pone.0290984)
Supplement: S2 File — (DOCX) [file pone.0290984.s003.docx]

**Application for ethical review – Appendix 1**

**Description of the research project**

**2. Type of research**

**2.1. In what ways does the project involve research according to §§ 3-4 of the ethical review law?**

**3 § 1 The research will collect sensitive personal data.**

3 § 2 The research will collect personal data on criminal offenses.

4 § 1 The research involves a physical intervention on a research subject.

**4 § 2 The research is conducted using a method intended to physically or mentally influence the research subject, or the research involves an obvious risk of harming the research subject.**

4 § 3 The research involves studies on biological material taken from a living human and that can be traced back to that person.

4 § 4 The research involves a physical intervention on a deceased human.

4 § 5 The research involves studies on biological material taken from a deceased human and that can be traced back to that person. The research does not fall within the scope of the ethical review law.

**2.2. [Regarding 3 § 1] Specify the type of sensitive personal data that will be processed in the project.**

Race or ethnic origin

Political opinions

Religious or philosophical beliefs

Membership in a trade union

**Health**

A person’s sex life or sexual orientation

Genetic data

**3. Purpose and research questions**

**3.1. Write a popular science summary of the research project (max 300 words).**

Being too physically and/or mentally aroused is a common cause and maintaining factor in insomnia. Both insomnia and ADHD are associated with high arousal levels, and many people with ADHD also have sleep problems. Calming down one’s breathing rate can be a way to calm down and facilitate falling asleep, but this can be difficult to achieve without support. The SOMNOX sleep robot provides physical and auditory guidance to calm down the user’s breathing rate. The advantage of the sleep robot is that it is intuitively easy to use and does not require that one is able to pay attention in different exercises like in Cognitive Behavioral Therapy. The sleep robot can also be an alternative where a so-called weighted blanket is not suitable for medical reasons.

Study 1…[Not translated]

Study 2…[Not translated]

In Study 3, we wish to investigate the effects of the sleep robot in adults with ADHD and insomnia. The study will be a within-group study with an AB design (n=20). Individual exploratory interviews will be conducted with the participants after the intervention has been completed.

The project is a collaboration between DigitalWell Arena and the Department of Social and Psychological Studies (both at Karlstad University). There are no ties to the manufacturer of SOMNOX.

**3.2. What is the scientific purpose of the project?**

The scientific purpose of the project is to investigate whether the use of the sleep robot has an effect on participants' symptoms of insomnia and sleep-related arousal. It is hypothesized that the active mechanism of the sleep robot is the calming effect resulting from slower breathing rates, which could potentially improve sleep in individuals with sleep-arousing conditions. The project aims to investigate whether the use of the sleep robot has an effect on participants' symptoms of insomnia, sleep-related arousal, anxiety, depression, and in Study 3, the degree of ADHD symptoms.

**3.3. What are the scientific research questions?**

1. Study 1…[Not translated]

2. Study 2…[Not translated]

3. Study 3, quantitative and qualitative data: Is the use of THE SOMNOX sleep robot associated with reduced sleep difficulties (severity of insomnia), as well as reduced symptoms of anxiety, depression and ADHD in individuals diagnosed with ADHD and insomnia compared to baseline? How do the participants experience the sleep robot?

**4. Method**

**4.1. Describe the method, including the procedure, technique, or treatment**

The research group has four sleep robots, which we want to investigate the effects of on sleep in adults with insomnia in Study 1 and 2, and in adults with ADHD and insomnia in Study 3.

Study 1…[Not translated]

Study 2…[Not translated]

In Study 3, the effects of the sleep robot on adults with ADHD and insomnia will be investigated. In Study 1, participants are instructed to lie in bed and hug the sleep robot for up to half an hour or until they experience the desired effect before going to sleep. Participants are free to adjust this to their own needs, for example, by sitting up or using the robot for a shorter or longer period. The instruction for participants in Studies 2 and 3 will depend on the results from Study 1. The instruction will be more stringent than in Study 1 and apply to all participants.

Recruitment will take place via social media and ads on campus at Karlstad University for the first two studies and via social media and user organizations for Study 3. Possible participants in the study will be contacted by phone and given more information about the study through a website. Those who are interested in participating in the study will then fill out two screening questionnaires for insomnia and pre-sleep arousal (Insomnia Severity Index and Pre-Sleep Arousal Scale). Those who score above the threshold will undergo further screening by phone (The Duke Structured Interview for Sleeping Disorders; Mini International Neuropsychiatric Interview). Participants will have the opportunity to ask questions about the study during the assessment interview, which is estimated to take 1.5 hours for eligible participants (shorter for those who are progressively found to be ineligible). Participants who wish to participate in the study will give informed consent in writing when receiving the sleep robot in Study 1 and 3, and at the first measurement point in Study 2. The instruction on how the sleep robot works takes up to one hour. With the support of a member of the research group, participants download an application on their phone (SOMNOX official app) and familiarize themselves with the robot's different functions. At the end of the intervention phase, the sleep robot is handed over to a member of the research team on site at Karlstad University. By agreement, a member of the research group can retrieve the sleep robot as needed to ensure that we receive it on time before the next participant takes over.

The treatment will be evaluated in several ways. Firstly, participants will complete a sleep diary daily during the baseline, intervention, and the week following the intervention in Study 1 and 3, and only the week before and after the intervention in Study 2. This is estimated to take a maximum of 5 minutes per day. The sleep diary will also include questions about how the participant has used the sleep robot. Secondly, participants in Study 1 will wear an activity bracelet every night during the study to obtain as objective measures as possible of sleep onset and duration by measuring their movements during the night. Thirdly, participants will complete the following questionnaires at pre-, post-, and follow-up measurements on sleep as well as symptoms of anxiety and depression in Study 1 and 2: Insomnia Severity Index (ISI: Bastien et al., 2001); Pre-sleep Arousal Scale (PSAS: Nicassio et al., 1985); Hospital Anxiety and Depression Scale (HADS: Zigmond & Snaith, 1983). In addition, participants in Study 3 will also complete the Adult ADHD Self-Report Scale (ASRS: Kessler et al., 2005). Completing these questionnaires is estimated to take 15-20 minutes per occasion and will be conducted on a total of three occasions during the study in Study 1 and 3, and on four occasions in Study 2.

Following the post-measurements in Study 1 and 3, an individual exploratory interview will be conducted where participants will have the opportunity to express their experiences with the sleep robot. The interview will also include questions about how their sleep difficulties have developed and what other treatment interventions the participants have previously tried. Based on previous post-intervention interviews with a similar question format, it is estimated that the interview will take approximately 15-45 minutes depending on how much the participants have to share.

**4.2. Describe in what way the method differs from clinical routine or conventional treatment.**

When treating sleep difficulties, the recommendation is to always start with non-pharmacological treatments due to the risk of side effects and dependence with certain medicines (SBU, 2010). However, prescribed medicines for sleep problems are increasing for both children and adults. Melatonin is a sleep hormone that naturally occurs in the body. The prescription of melatonin in Sweden has increased significantly since it was introduced as a medication in 2008, with an increase of 40% for patients with ADHD between 2015 and 2016 alone, according to the National Board of Health and Welfare (2017). The increase may be due to more people being diagnosed with ADHD, but also because melatonin now is prescribed without a license from the Swedish Medical Products Agency. Prescribing melatonin costs the healthcare system a lot of money.

Several techniques for winding down have been shown to have beneficial effects on symptoms of insomnia. Psychological treatments in the form of Cognitive Behavioral Therapy for insomnia (CBT-I) is the gold standard treatment of insomnia. CBT-I has only recently begun to be evaluated for people with neuropsychiatric difficulties and sleep problems (Jernelöv et al., 2019). Psychological treatments are relatively time-consuming and require more therapists to be trained (SBU, 2010). In addition, early findings suggest that existing approaches may need to be tailored for this patient group. If sleep robots have positive effects on sleep, they may provide an alternative to CBT-I for people who are deemed unable to benefit from such treatment. Sleep robots can also provide an alternative to existing medical devices such as various weighted blankets when they are not suitable due to medical reasons (e.g., overweight, breathing difficulties), or when the conventional treatments are ineffective.

**4.3. Describe previous experiences (personal and/or others’) with the used procedure, technique, or treatment.**

The research group consists of experts in sleep (Annika Norell-Clarke), digital health technology (Erik Wästlund), neuropsychology (Siri Jakobsson Støre), and treatment studies (Maria Tillfors & Annika Norell-Clarke). All of these areas of expertise are needed to carry out and evaluate the effects of the SOMNOX sleep robot for individuals with insomnia, with and without ADHD.

There are no previous studies conducted on the SOMNOX sleep robot. The company's own research appears to be unsystematic and only of qualitative nature. There are no affiliations with the manufacturer of SOMNOX. The research group has been in contact with key persons in a municipality in Värmland, Sweden, who have used the SOMNOX sleep robot within the elderly care. The experiences there were that the sleep robot seemed to have a calming effect on those who did try it. The biggest challenge seems to have been the technical aspect since the sleep robot, according to the company, has optimal effect if one downloads an app and adjusts the settings to the individual who uses the sleep robot.

**5. Timeline**

**5.1. Expected start date of the project:**

February 2021

**5.2. Expected end date of the project:**

June 2023

**5.3. Timeline for the different parts included in the project:**

Study 1…[Not translated]

Study 2…[Not translated]

Recruitment to Study 3 is planned to start during fall of 2022, with data collection until the summer of 2023.

**6. Data collection**

**6.1. Describe the data collection and the nature of the data.**

People who are interested in participating in the studies will first undergo a shorter screening, step 1, which will consist of the questionnaires the Insomnia Severity Index (ISI: Bastien et al., 2001), and the Pre-Sleep Arousal Scale (PSAS: Nicassio et al., 1985). Individuals who have elevated levels of ISI (above 8, indicating symptoms at clinical levels) and PSAS (above 10 on the somatic scale) will undergo the following structured interviews, step 2, administered by a licensed psychologist or psychology student under the supervision of a licensed psychologist:

- The Duke Structured Interview for Sleeping Disorders (DSISD: Carney et al., 2009), to ensure that individuals included in the study have a diagnosis of insomnia, and not symptoms that can be better explained by another untreated sleep disorders.
- Mini International Neuropsychiatric Interview (M.I.N.I.: Sheehan et al., 1998), to exclude that participants meet the criteria for any other psychiatric diagnosis.

The exclusion criteria are ongoing alcohol, drug, or medication abuse during the past three months, psychosis, severe depression, or current bipolar symptoms, and suicidal ideation. Any ongoing medication treatment for ADHD and/or sleep difficulties must be well-adjusted (optimal dose for at least two months).

The treatment is evaluated in several ways. Firstly, in Study 1 and 3 by participants filling out a sleep diary on a daily basis during the baseline, intervention, and the week following the intervention. In Study 2, participants complete the sleep diary only the week before and the week following the intervention. This is estimated to take a maximum of 5 minutes per day. The sleep diary also includes a few questions about how the participant has used the sleep robot. Secondly, participants in Study 1 will wear an actigraph every night during the study to obtain as objective measurements as possible of the sleep onset and duration by measuring their movements during the night. Thirdly, participants in all three studies will complete the following questionnaires at pre-, post-, and follow-up measurements for sleep, anxiety, and depression symptoms:

- Insomnia Severity Index (ISI: Bastien et al., 2001), which is the "gold standard" questionnaire for evaluating insomnia treatments, and which measures the degree of insomnia symptoms.
- Pre-sleep Arousal Scale (PSAS: Nicassio et al., 1985), which measures the degree of physical and mental arousal.
- Hospital Anxiety and Depression Scale (HADS: Zigmond & Snaith, 1983), which measures the degree of anxiety and depression symptoms.

In addition to the measurement tools mentioned above, participants in Study 3 will also complete the Adult ADHD Self-Report Scale (ASRS: Kessler et al., 2005), which measures the degree of ADHD symptoms.

In connection with the follow-up in Study 1 and 3, individual exploratory interviews will be conducted with participants to give them the opportunity to disclose their experiences with the sleep robot. The interview also includes questions about how their sleep problems have developed and what other treatment interventions the participants have tried before. Based on previous post-intervention interviews with a similar question format, it is estimated that the interview will take about 15-45 minutes depending on how much the participants have to say. The interviews will be transcribed by university employees.

A number of demographic factors will be collected to describe the participants in a comprehensive manner. These include age, gender (male, female, non-binary, other), marital status, occupation, education, ethnicity, number and age of children, as well as psychiatric and somatic diagnoses.

Regarding the clinical variables, the primary outcome measure is the Insomnia Severity Index. Secondary outcome measures are variables from the sleep diary and actigraphs, as well as the PSAS and the HADS questionnaires (as well as the ASRS in Study 3).

**6.2. Describe the statistical basis for the study population/ sample size:**

Study 2…[Not translated]

The plan is to recruit 20 participants to Study 3. This study is a within-subjects study with an AB design, and 20 participants are considered sufficient to achieve statistical power.

**6.3. How will the research procedures be documented?**

The individual interviews conducted after the intervention in Study 1 and 3 will be recorded with a tape recorder. Answers to questions from the structured diagnostic interviews will be documented on the corresponding forms using pen and paper.

**6.4. How will collected data be handled and stored?**

Study 2…[Not translated]

Data will be processed and stored in accordance with the General Data Protection Regulation (GDPR). The data will be pseudonymized, and the code key with identifying personal information (which code corresponds to which personal information) will be stored separately from the data. No unauthorized person will have access to the material. In accordance with the *arkivlagen* [Archives Act], data will be saved for 10 years after the completion of the study and then destroyed.

**7. Ethical considerations**

**7.1. What risks can participation in the research project pose to research participants?**

An ethical consideration concerns the individual's experience of intrusion of privacy when sensitive questions about personal information, mental symptoms, and well-being are asked. The questionnaires used have been used in several studies without any known problems arising due to the nature of the questions. The individual exploratory interviews will be audio recorded. Generally, our experience is that neither audio recording nor interviews are perceived as unpleasant. The project will comply with the requirements of information, consent, confidentiality, and utilization.

The research material (questionnaires, audio recordings, and statistics from actigraphs) is stored on two purposefully encrypted USB drives that are locked in cabinets accessible only to the project leader. A numerical code and key code with information about the name and personal identification number are established for each participant. The key code is stored in a locked archive cabinet in another room. Only researchers working on the study will be able to access the results for individual participants. Results are presented at both group and individual levels (quantitative and qualitative data). In studies where the results are presented at the individual level (such as quotes from interviews), there is a risk that participants will be recognizable. Therefore, we will de-identify data and be cautious about associating individual results with specific quotes that could reveal participants' anonymity. As such, the compilation is carried out in a way in which individual participants cannot be identified.

An ethical dilemma of clinical relevance, especially regarding Study 3, is that the study offers a treatment that participants cannot continue after the study, even if the sleep robot proves to have a positive effect on patients' symptoms. However, after the study, participants will receive information about standard treatment for neuropsychiatric difficulties and insomnia, and advice on how to seek such help. It is also theoretically possible that the sleep robot has a long-term effect even after discontinuation, for example, if participants have learned relaxation strategies.

Some participants may be disappointed if the intervention with the sleep robot does not produce the expected effect. To minimize this risk, the research group will inform all participants about this possible outcome. Some participants may also experience worsening symptoms during the study, either regarding their sleep symptoms or other psychiatric symptoms. The research group consists of three psychologists, including a specialist psychologist [PsyD], who are experienced in handling such situations and will help participants manage their emotions and symptoms in the moment, as well as refer participants to the appropriate health care agency if necessary. The research group assesses the risk of deterioration due to the sleep robot to be very low.

People interested in participating in the study but who meet one or more exclusion criteria and are therefore not allowed to participate may perceive this negatively. There is a risk that those who are excluded from the study will not seek other, more appropriate help, which is why all those who are excluded will be actively guided to where they can seek more adequate help.

Regarding infection safety, the sleep robot has a cover that is washed between different participants' use of the robot. Screening is mainly done by phone, alternatively in a special room at the university with plexiglass between the researcher and the participant if there is a special request for a physical meeting. There is access to hand washing and hand sanitizer at physical meetings, which in principle only occur when picking up and returning the sleep robot. Participants with symptoms of COVID-19 are urged to inform the research group as soon as possible. In cases where that happens when a participant has the sleep robot, the group will consult with the security chief at Karlstad University regarding appropriate handling of the sleep robot. A sleep robot that has been with a participant with symptoms will not be released again until an expert confirms that it would be safe to do so.

**7.2. What benefit can participation bring to the research participants included in the research project?**

Relaxation has general beneficial effects on health, both physical and mental. Both insomnia and ADHD are associated with too high arousal levels. Calming down one's breathing rate can be one way to calm down and facilitate sleep, but this can be difficult to do on one's own without support. In the project, participants will have access to cutting-edge research in the field of insomnia that can improve healthcare for people with sleep difficulties in general, but perhaps especially those with comorbid neuropsychiatric difficulties and insomnia, which represent a neglected group in sleep research. It is also possible that the sleep robot will have long-term positive effects on participants' sleep after short-term use, for example, by teaching participants strategies to wind down.

**7.3. Make an assessment of the balance between the risks and benefits of the project.**

All research studies require some time and involve some risks for the participants. The time frame for the participants in our project is relatively short, and the potential benefits of the study are considered to outweigh the possible risks.

**7.4. Describe how the project has been designed to minimize the risks to the research subjects.**

The research team gradually examines the effects and ways of use on a few individuals in a very controlled manner in Study 1, to include more participants and have better-supported instructions for participants in Study 2, and finally, in Study 3, to include more vulnerable individuals and examine them more closely. All participants in all three studies will receive the intervention. The research team consists of three psychologists (including a specialist psychologist). Participants are encouraged to contact us with any questions or in case of a deterioration in their conditions, and in such cases, the research team is prepared to handle the situation temporarily and to guide participants to adequate help.

**7.5. Identify and specify any ethical problems (disadvantages/advantages) that may arise in a broader perspective through the research project.**

If the sleep robot shows good effects on sleep for the participants in our project, it may become a treatment alternative in healthcare for people with neuropsychiatric difficulties and comorbid sleep disorders who may not be able or willing to undergo CBT-I treatment, and where existing medications and medical devices do not work or cannot be tested due to medical reasons. The results from our project may also guide future studies on sleep robots with other types of sleep disorders and (neuro)psychiatric conditions.

**8. Research participants**

**8.1. How are research participants selected?**

Study 1…[Not translated]

Study 2…[Not translated]

The research group plans to recruit 20 individuals with ADHD and insomnia to Study 3. Recruitment will happen through social media and interest organizations.

**8.2. How many research participants will be included in the research project?**

Study 1…[Not translated]

Study 2…[Not translated]

20 participants in Study 3.

**8.3. What selection criteria will be used for inclusion?**

Individuals interested in participating in the research project will first undergo a brief screening, step 1, which will consist of the self-assessment questionnaires Insomnia Severity Index (ISI) and Pre-Sleep Arousal Scale (PSAS). Those with elevated levels (above 8 on the ISI and above 10 on the somatic scale of the PSAS) will be contacted to undergo two structured telephone interviews:

- The Duke Structured Interview for Sleeping Disorders (DSISD), to confirm that individuals included in the study have a diagnosis of insomnia, but not insomnia symptoms that can be better explained by another untreated sleep disorder.
- Mini International Neuropsychiatric Interview (M.I.N.I.), to exclude participants who meet any of the exclusion criteria.

**8.4. What selection criteria will be used for exclusion?**

Study 1…[Not translated]

Study 2…[Not translated]

Study 3 has the following exclusion criteria: ongoing alcohol, drug or medication abuse in the last three months, psychosis, severe depression or current bipolar symptoms, as well as suicidality. Any ongoing medication for ADHD and/or sleep difficulties must be well-adjusted (optimal dose for at least two months).

**8.5. What is the relationship between researchers and research participants?**

Study 1…[Not translated]

Study 2…[Not translated]

Study 3: No relationships between researchers and research participants.

**8.6. What insurance coverage is available for the research participants participating in the research project?**

Patient injury insurance applies.

**8.7. Describe the preparedness to handle unexpected findings or events during the research process that could jeopardize the safety of research participants.**

The research team consists of three psychologists, including a specialist psychologist, who are experienced in handling unexpected situations both clinically and in treatment studies. Currently, the possibility of contracting COVID-19 is a significant unexpected event that may require measures in the project. Participants with symptoms of COVID-19 are encouraged to inform the research group as soon as possible. In cases where the sleep robot is located with such a participant, the group will consult with the security manager at KAU regarding the appropriate handling of the sleep robot. A sleep robot that has been with a participant with symptoms will not be released again until an expert confirms that it would be safe to do so.

**8.8. Will research participants receive financial compensation or other benefits?**

No.

**9. Information and consent**

**9.1. Will research participants be informed about the research project and asked if they want to participate or not?**

Yes.

**9.1.1. [If Yes to 9.1] How, when (at what stage), and by whom will the research subjects be informed and asked to participate?**

Written information about the study is distributed/posted on campus at Karlstad University and via social media in the first two sub-studies, and additionally through user organizations in Sub-study 3. Those who show interest in the study are called and given more information about the study. Everyone interested in participating in the study receives additional written information about the study through a website. The research subjects will also undergo a longer telephone interview where they will have the opportunity to ask questions to the interviewing licensed psychologist (possibly a psychology student under the supervision of a licensed psychologist) about the study. They are also given time to consider their participation before providing their written consent to participate in the study at a later time.

**9.2. Will children under 18 years of age be included in the research project?**

No

**9.3. Will research subjects whose consent cannot be obtained due to illness, mental disorder, weakened health condition, or any other similar circumstances be included in the research project?**

No

**10. Registry data**

**10.1. Will the project request data from an existing registry?**

No.

**11. Animal experimentation results**

**11.1. Are there any relevant results from animal experiments?**

Not applicable.

**12. Reporting of results**

**12.1. How is access to data guaranteed for the principal investigator and participating researchers?**

All researchers in the research group have full access to the data. Only researchers working on the study will be able to access the results for individual participants. The research group consists of: Annika Norell-Clarke (Associate Professor of Psychology, licensed psychologist), Maria Tillfors (Professor of Psychology, licensed psychologist), Erik Wästlund (Associate Professor of Psychology), and Siri Jakobsson Støre (PhD student in Psychology, specialist psychologist).

**12.2. Who is responsible for data processing and written reporting of the results?**

The main responsible are Associate professor Annika Norell-Clarke, PhD student Siri Jakobsson Støre, professor Maria Tillfors and Associate professor Erik Wästlund.

**12.4. In what way are research participants' rights to integrity guaranteed when the material is published?**

In studies where the results are presented on an individual level (e.g. quotes from interviews), there is a risk that participants will be recognizable. The research group will therefore de-identify data and be cautious about linking individual results with specific quotes that could reveal the anonymity of participants.

Study 2…[Not translated]

**13. Economic conditions**

**13.1. Report any financial agreements with donors or other financiers (name and amount).**

Not applicable.

**13.2. Report the research manager's, the main responsible researcher's and the participating researchers' own financial interests.**

No personal financial interests.
