# Supplementary material for: A robot intervention for adults with ADHD and insomnia–A mixed-method proof-of-concept study
Source: PLoS One. 2023 Sep 1;18(9):e0290984. doi: 10.1371/journal.pone.0290984 (PMC10473504; doi:10.1371/journal.pone.0290984)
Supplement: S3 File — (PDF) [file pone.0290984.s004.pdf]

Ansökan om etikprövning – *Bilaga 1*
Beskrivning av forskningsprojektet

2. Typ av forskning

2.1. På vilket eller vilka sätt handlar projektet om forskning enligt 3-4 §§ etikprövningslagen?

- [x] 3 § 1 Forskningen kommer att samla in känsliga personuppgifter.
- [ ] 3 § 2 Forskningen kommer att samla in personuppgifter om lagöverträdelser.
- [ ] 4 § 1 Forskningen innebär ett fysiskt ingrepp på en forskningsperson.
- [x] 4 § 2 Forskningen utförs enligt en metod som syftar till att påverka forskningspersonen fysiskt eller psykiskt, eller så innebär forskningen en uppenbar risk att skada forskningspersonen.
- [ ] 4 § 3 Forskningen avser studier på biologiskt material som har tagits från en levande människa och kan härledas tillbaka till denna människa.
- [ ] 4 § 4 Forskningen avser ett fysiskt ingrepp på en avliden människa.
- [ ] 4 § 5 Forskningen avser studier på biologiskt material som tagits från en avliden människa och kan härledas tillbaka till denna människa.

- [ ] Forskningen faller inte under etikprövningslagens tillämpningsområde.

Forskningsperson: De levande människor som forskningen avser.

Personuppgifter: All slags information som direkt eller indirekt kan hänföras till en fysisk person som är i livet.

Känsliga personuppgifter: Känsliga personuppgifter är uppgifter som avslöjar ras eller etniskt ursprung, politiska åsikter, religiös eller filosofisk övertygelse, medlemskap i fackförening, hälsa, en persons sexualliv eller sexuella läggning, genetiska uppgifter och biometriska uppgifter som entydigt identifierar en person.

Personuppgifter om lagöverträdelser: Personuppgifter om lagöverträdelser som innefattar brott, domar i brottmål, straffprocessuella tvångsmedel eller administrativa frihetsberövanden.

2.2. [Om 3 § 1] **Ange vilken typ av känsliga personuppgifter som kommer behandlas i projektet.**

- [ ] ras eller etniskt ursprung
- [ ] politiska åsikter
- [ ] religiös eller filosofisk övertygelse
- [ ] medlemskap i fackförening
- [x] hälsa
- [ ] en persons sexualliv eller sexuella läggning
- [ ] genetiska uppgifter
- [ ] biometriska uppgifter som entydigt identifierar en person.

Personuppgifter: All slags information som direkt eller indirekt kan hänföras till en fysisk person som är i livet.

Känsliga personuppgifter: Känsliga personuppgifter är uppgifter som avslöjar ras eller etniskt ursprung, politiska åsikter, religiös eller filosofisk övertygelse, medlemskap i fackförening, hälsa, en persons sexualliv eller sexuella läggning, genetiska uppgifter och biometriska uppgifter som entydigt identifierar en person. Enligt definition i EU:s Dataskyddsförordning.

OBS! Om inga känsliga personuppgifter kommer behandlas i projektet så behöver frågan inte besvaras.

3. Syfte och frågeställningar

3.1. Skriv en populärvetenskaplig sammanfattning av forskningsprojektet (max 300 ord).

Beskriv projektet på ett sammanfattande vis. Tänk på att texten ska kunna förstås av personer som inte har vetenskaplig kompetens. Undvik därför terminologi som kräver specialkunskaper.

Att vara alltför fysiskt och/eller mentalt uppvarvad är en vanlig orsak till och en vidmakthållande faktor vid insomni. Både insomni och ADHD är förknippade med för hög uppvarvningsnivå och många personer med ADHD har även sömnproblem. Att lugna ned sin andningstakt kan vara ett sätt att lugna ned sig och underlätta insomningen men det kan vara svårt att få till utan stöd. SOMNOX sömnrobot ger fysisk och auditiv vägledning för att lugna ned andningstakten. Fördelen med sömnroboten är att den är intuitivt enkel att använda och att det inte krävs att man lär sig övningar eller har tillräckligt med förmåga att fokusera för att exempelvis ta till sig KBT-baserade "sömnskolor". Sömnroboten kan också vara ett alternativ där ett s.k. tyngdtäcke inte är aktuellt på grund av medicinska skäl.

Delstudie 1 är en explorativ single-case studie (n=4), med en s.k. A (baslinje) B (intervention) design, med vuxna personer med insomni. Deltagarna fyller i en sömndagbok under baslinjen, under samt efter interventionen, totalt 6 veckor. Deltagarna fyller också i före-och eftermätningar i direkt anslutning till interventionen, samt vid uppföljning fyra veckor senare. Deltagarna kommer även att ha på sig ett aktivitetsarmband varje natt under studien. Vidare genomförs individuella explorativa intervjuer med deltagarna efter genomförd intervention.

Delstudie 2 är en randomiserad kontrollerad studie (n=32) med vuxna personer med insomni. Deltagarna fyller i före-och eftermätningar i direkt anslutning till interventionen samt vid uppföljning efter fyra veckor. Deltagarna fyller också i en sömndagbok veckan innan och veckan efter interventionen.

I delstudie 3 önskar vi undersöka effekterna av sömnroboten på vuxna personer med ADHD och insomni. Studien kommer att vara en inomgruppsstudie med en AB-design (n=20). Också här genomförs individuella explorativa intervjuer med deltagarna efter genomförd intervention.

Projektet är ett samarbete mellan DigitalWell Arena och psykologiämnet (båda vid Karlstads universitet). Inga bindningar till tillverkaren av SOMNOX finns.

3.2. Vad är det vetenskapliga syftet med projektet?

Beskriv det övergripande syftet med projektet. Redogör för vad det är för forskningsproblem som projektet ska behandla och vilka avgränsningar som gjorts.

Den verksamma mekanismen hos sömnroboten antas vara nedvarvning via långsammare andningstakt, vilket leder till den övergripande hypotesen att den skulle kunna förbättra sömnen hos personer med sömnstörande uppvarvning. Syftet med projektet är att undersöka om användning av sömnroboten har en effekt på deltagarnas insomnisymtom, samt effekt på sömnstörande uppvarvning, symtom på ångest och depression, och för Delstudie 3 också graden av ADHD-symtom.

3.3. Vilka är de vetenskapliga frågeställningarna?

Ange klart och tydligt den eller de vetenskapliga frågeställningarna i projektet.

1. Delstudie 1 kvantitativa och kvalitativa data: Leder användning av SOMNOX sömnrobot till minskade sömnsvårigheter (svårighetsgrad av insomni) samt minskning av ångest- och depressionssymtom hos personer med insomnidiagnos och sömnstörande uppvarvning jämfört med baslinjen? Hur upplever deltagarna att använda sömnroboten? Hur länge måste de använda sömnroboten innan den eventuellt har en effekt? Överensstämmer statistiken från aktivitetsarmbanden med deltagarnas självskattning av sömnen?
2. Delstudie 2 kvantitativa data: Leder användning av SOMNOX sömnrobot till minskade sömnsvårigheter (svårighetsgrad av insomni) samt minskning av ångest- och depressionssymtom och sömnstörande uppvarvning hos personer med insomnidiagnos och sömnstörande uppvarvning jämfört med väntelistekontroll?
3. Delstudie 3 kvantitativa och kvalitativa data: Leder användning av SOMNOX sömnrobot till minskade

sömnsvårigheter (svårighetsgrad av insomni) samt minskning av ADHD-, ångest- och depressionssymtom och sömnstörande uppvarvning hos personer med diagnosticerad ADHD och insomni jämfört med baslinjen? Hur upplever deltagarna att använda sömnroboten?

4. Metod

4.1. Redogör för metod inkl. proceduren, tekniken eller behandlingen.

Det ska framgå hur projektet planeras att genomföras. Beskriv insamlade datas karaktär och ange hur datas tillförlitlighet ska säkerställas.

Om enkäter och intervjuer ingår ska tillvägagångssätt, frågornas innehåll och hur slutsatser dras beskriva. Bifoga enkäter och skattningsskalor.

För medicinsk forskning ska anges t.ex. typer av ingrepp, mätmetoder, antal besök, tidsåtgång, doser, och administrationssätt för eventuella läkemedel.

Om projektet är ett samarbete med utlandet ska det tydligt framgå vilken del av forskningen som utförs i Sverige och vilken del av forskningen som utförs utanför Sverige. Det är endast forskning som ska utföras i Sverige som Etikprövningsmyndigheten kan pröva.

Forskargruppen har fyra sömnrobotar. Forskargruppen önskar undersöka effekten av roboten på sömnen hos vuxna personer med insomni i Delstudie 1 och 2; hos vuxna personer med ADHD och insomni i Delstudie 3. Delstudie 1 är en explorativ single-case studie (n=4), med en s.k. A (baslinje) B (intervention) design, med vuxna personer med insomni. Delstudie 2 är en randomiserad kontrollerad studie (n=32) med vuxna personer med insomni. I delstudie 3 önskar vi undersöka effekterna av sömnroboten på vuxna personer med ADHD och insomni. Instruktionen till deltagarna i Delstudie 1 är att de förslagsvis ligger i sängen och kramar om sömnroboten i upp till en halvtimme, eller tills önskad effekt uppkommit, i anslutning till sänggåendet. Deltagarna är fria till att anpassa detta till egna behov, om man till exempel föredrar att sitta upp, eller önskar använda roboten kortare eller längre tid. Instruktionen till deltagarna i Delstudie 2 och 3 beror på vad som framkommer i Delstudie 1. Instruktionen kommer att vara mer stringent jämfört med första delstudien och gälla för samtliga deltagare.

Rekrytering kommer ske via sociala medier och annonser på campus på Karlstads universitet till de två första delstudierna; via sociala medier och brukarorganisationer till Delstudie 3. Möjliga deltagare i studien kommer att kontaktas via telefon, och sedan få mer information om studien genom en websida. De som är intresserade av att delta i studien kommer sedan att få fylla två screeningformulär för insomnia och sömnstörande uppvarvning (Insomnia Severity Index och Pre-Sleep Arousal Scale). De som skårar över gränsvärden kommer att genomgå ytterligare en screening via telefon (The Duke Structured Interview for Sleeping Disorders; Mini International Neuropsychiatric Interview). Här får deltagarna möjlighet att ställa frågor om studien. Bedömningssamtalet beräknas ta 1,5 timmar för aktuella deltagare (kortare för de som successivt visar sig inte vara aktuella). De som önskar delta i studien ger informerat samtycke skriftligt i samband med utlämningen av sömnroboten i Delstudie 1 och 3, och i samband med första mätpunkten för deltagarna i Delstudie 2. Instruktion i hur sömnroboten fungerar tar upp mot en timme. Med stöd från medlem i forskargruppen laddar deltagarna ner en applikation på telefonen (SOMNOX officiella app) och bekantar sig med robotens olika funktioner. Vid interventionsfasens slut lämnas sömnroboten till medlem i forskargruppen på plats på Karlstads universitet; vid överenskommelse kan medlem i forskargruppen hämta sömnroboten vid behov, detta för att säkerställa att vi får in den i tid innan nästa deltagare ska ta över den.

Utvärdering av behandlingen sker på flera sätt. För det första utvärderas den genom att deltagarna fyller i en så kallad sömndagbok dagligen under baslinjen, interventionen, samt veckan efter interventionen i Delstudie 1 och 3, endast veckan innan och veckan efter interventionen i Delstudie 2. Detta beräknas ta max 5 minuter per dag. Sömndagboken innehåller också frågor om hur deltagaren har använt sömnroboten. För det andra kommer deltagarna i Delstudie 1 att ha på sig ett aktivitetsarmband varje natt under studien för att vi ska få så objektiva mått som möjligt på insomningstid och sömnlängd genom att mäta deltagarnas rörelser under natten. För det tredje fyller deltagarna i följande frågeformulär vid före-, efter- och uppföljningsmätningarna på sömn samt symtom på ångest och depression i Delstudie 1 och 2: Insomnia Severity Index (ISI: Bastien et al., 2001); Pre-sleep Arousal Scale (PSAS: Nicassio et al., 1985); Hospital Anxiety and Depression Scale (HADS: Zigmond & Snaith, 1983). Utöver detta fyller deltagarna i Delstudie 3 även i: Adult ADHD Self-Report Scale (ASRS: Kessler et al., 2005). Ifyllande av dessa formulär beräknas ta 15–20 minuter per tillfälle och genomförs vid totalt tre tillfällen under studiens gång i Delstudie 1 och 3; vid totalt fyra tillfällen i Delstudie 2.

I anslutning till eftermätningarna i Delstudie 1 och 3 genomförs en individuell explorativ intervju där deltagarna får möjlighet att föra fram sina upplevelser kring sömnroboten. Intervjun innehåller också frågor om hur sömnbesvären har utvecklat sig och vilka andra behandlingsinterventioner deltagarna har testat tidigare. Utifrån tidigare post-interventionsintervjuer med liknande frågeupplägg görs bedömningen att intervjun tar ungefär 15-45 minuter beroende på hur mycket deltagarna har att berätta.

4.2. **Redogör för på vilket sätt metoden skiljer sig från klinisk rutin eller den ordinarie behandlingen.**

Ange vad som avviker eller tillkommer med anledning av forskningsprojektet.

Vid behandling av sömnsvårigheter är rekommendationen att alltid börja med ickefarmakologisk behandling på grund av risken för biverkningar och beroende vid vissa preparat (SBU, 2010), men förskrivning av läkemedel mot sömnbesvär ökar till både barn och vuxna. Melatonin är ett sömnhormon som finns naturligt i kroppen. Förskrivningen av melatonin i Sverige har ökat kraftigt sedan det introducerades som läkemedel i 2008, en ökning på hela 40 procent för patienter med ADHD bara mellan 2015 och 2016, enligt Socialstyrelsen (2017). Ökningen kan bero på att allt fler diagnosticeras med ADHD men också på att det nu går att skriva ut melatonin utan licens från Läkemedelsverket. Förskrivning av melatonin kostar hälso- och sjukvården mycket pengar.

Strategier för att varva ned har evidens vid insomni gällande ett flertal tekniker. Psykologiska behandlingsmetoder i form av kognitiv beteendeterapi (KBT-I) är golden standard vid behandling av insomni. KBT-I har för personer med neuropsykiatriska svårigheter och sömnsvårigheter endast börjat utvärderas (Jernelöv et al., 2019). Psykologiska behandlingsmetoder är generellt relativt tidskrävande och förutsätter att fler terapeuter utbildas (SBU, 2010). Dessutom tyder tidiga fynd på att de befintliga uppläggen kan komma att behöva specialsys för denna patientgrupp. Om sömnrobotar ger positiv effekt på sömnen kan den utgöra ett alternativ till KBT-I för personer som bedöms inte kunna tillgodogöra sig en sådan behandling. Sömnroboten kan också utgöra ett alternativ till de befintliga medicintekniska produkterna så som olika tyngdtäcken där dessa inte är aktuella av medicinska skäl (övervikt, andningssvårigheter) eller inte ger optimal effekt, eller där medicinering inte fungerar.

4.3. **Redogör för tidigare erfarenheter (egna och/eller andras) av den använda proceduren, tekniken eller behandlingen.**

Redogör för vilken erfarenhet och kompetens som medverkande forskare har av att använda den procedur, teknik eller behandling om man planerar att använda i projektet. Redogör även för vilken erfarenhet som finns generellt eller globalt.

I forskargruppen ingår experter inom sömn (Annika Norell-Clarke), digital hälsoteknik (Erik Wästlund), neuropsykologi (Siri Jakobsson Störe) och behandlingsstudier (Maria Tillfors & Annika Norell Clarke). Samtliga expertkunskaper behövs för att genomföra och utvärdera effekterna av SOMNOX sömnrobot för personer med insomni, med och utan ADHD.

Det finns inga tidigare studier gjorda på SOMNOX sömnrobot. Företagets egen forskning verkar vara osystematisk och endast av kvalitativ art. Inga bindningar till tillverkaren av SOMNOX finns. Forskargruppen har haft kontakt med nyckelpersoner inom en kommun i Värmland som har använt SOMNOX sömnrobot inom äldreomsorgen. Erfarenheterna här har varit att sömnroboten verkar ha en lugnande effekt på de som har testat den. Den största utmaningen verkar ha varit det tekniska eftersom sömnroboten enligt bolaget har optimal effekt om man laddar ner en app och anpassar inställningarna till individen som ska använda sömnroboten.

5. Tidsplan

5.1. Förväntat startdatum för projektet:

Ange om möjligt, beräknad start av projektet.

Februari 2021

5.2. Förväntat slutdatum för projektet:

Ange om möjligt, beräknat slut av projektet.

Juni 2023

5.3. Tidsplan för de olika delar som ingår i projektet:

Delstudie 1 planeras genomföras under våren 2021, inkluderat intervjuer av deltagarna efter interventionen. Rekrytering till Delstudie 2 planeras ske under hösten 2021. Datainsamlingen förväntas pågå fram till sommaren 2022. Rekrytering till Delstudie 3 planeras påbörjas hösten 2022, och datainnsamlingen pågår efter planen fram till sommaren 2023.

Redogör för projektets tidsschema, hur de olika delarna förhåller sig till varandra, vid behov kan ett förklarande flödesschema bifogas.

6. Datainsamling

6.1. Redogör för datainsamling och datas karaktär.

Redogör för hur datainsamlingen ska gå till. Beskriv den data som ska samlas in och hur den ska samlas in.

Deltagare som är intresserade av att delta i forskningsstudien kommer först att få genomgå en kortare screening, steg 1, som kommer bestå av självskattningsformulären Insomnia Severity Index (ISI: Bastienet al., 2001) och Pre-Sleep Arousal Scale (PSAS: Nicassio et al., 1985). De personer som har förhöjda nivåer på ISI (över 8, som indikerar att symtom ligger på kliniska nivåer) och på PSAS (över 10 på den somatiska skalan), kommer att få genomgå följande strukturerade intervjuer som administreras av leg psykolog eller psykologstudent under handledning av leg psykolog:

- The Duke Structured Interview for Sleeping Disorders (DSISD: Carney et al., 2009), detta för att kontrollera att personer som inkluderas i studien har en diagnos av insomni, steg 2 men inte insomnisymtom som bättre kan förklaras av annan obehandlad sömnstörning.
- Mini International Neuropsychiatric Interview (M.I.N.I.: Sheehan et al., 1998), detta för att utesluta att deltagarna uppfyller kriterierna för några andra psykiatriska diagnoser i Delstudie 1 och Delstudie 2. Vad gäller Delstudie 3 är exklusionskriterierna: pågående alkohol-, drog- eller läkemedelsmissbruk under de senaste tre månaderna, psykos, svår depression eller en aktuell bipolär symtombild samt suicidalitet. Eventuell pågående läkemedelsbehandling för ADHD och/eller sömnsvårigheterna ska vara välinställd (optimal dos under minst två månader).

Utvärdering av behandlingen sker på flera sätt. För det första utvärderas den i Delstudie 1 och 3 genom att deltagarna fyller i en så kallad sömndagbok dagligen under baslinjen, interventionen, samt veckan efter interventionen. I Delstudie 2 fyller deltagarna i sömndagboken endast veckan innan och veckan efter interventionen. Detta beräknas ta max 5 minuter per dag. Sömndagboken innehåller också några frågor om hur deltagaren har använt sömnroboten. För det andra kommer deltagarna i Delstudie 1 att ha på sig ett aktivitetsarmband varje natt under studien för att vi ska få så objektiva mått som möjligt på insomningstid och sömnlängd genom att mäta deltagarnas rörelser under natten. För det tredje fyller deltagarna i alla tre delstudier i följande frågeformulär vid före-, efter- och uppföljningsmätningarna på sömn samt symtom på ångest och depression:

- Insomnia Severity Index (ISI: Bastien et al., 2001), som är ”golden standard” vid utvärdering av behandling för insomni och mäter graden av insomnisymtom.
- Pre-sleep Arousal Scale (PSAS: Nicassio et al., 1985), som mäter graden av fysisk och psykisk uppvarvning.
- Hospital Anxiety and Depression Scale (HADS: Zigmond & Snaith, 1983), som mäter graden av ångest- och depressionssymtom.

Utöver mätinstrumenten över fyller deltagarna i Delstudie 3 i följande formulär:

- Adult ADHD Self-Report Scale (ASRS: Kessler et al., 2005) som mäter graden av ADHD-symtom.

I anslutning till eftermätningen i Delstudie 1 och 3 genomförs en individuell explorativ intervju av deltagarna där de får möjlighet att föra fram sina upplevelser kring sömnroboten. Intervjun innehåller också frågor om hur sömnbesvären har utvecklat sig och vilka andra behandlingsinterventioner deltagarna har testat tidigare. Utifrån tidigare post-interventionsintervjuer med liknande frågeupplägg görs bedömningen att intervjun tar ungefär 15-45 minuter beroende på hur mycket deltagarna har att berätta. Transkribering av intervjuerna sker av anställda på universitetet.

Ett antal demografiska faktorer kommer att samlas in för att kunna beskriva deltagarna på ett fullödigt sätt. Det gäller följande: ålder, kön (man, kvinna, ickebinär, annat), civilstånd, sysselsättning, utbildning, etnicitet, antal och ålder på barn samt psykiatriska och somatiska diagnoser.

Vad gäller de kliniska variablerna är det primära utfallsmåttet Insomnia Severity Index. Sekundära utfallsmått är variabler från sömndagboken och aktivitetsarmbanden samt självskattningsformulären PSAS och HADS (samt ASRS i Delstudie 3).

6.2. Redogör för det statistiska underlaget för studiepopulationen/ undersökningsmaterialets storlek.

Redogör för de beräkningar och överväganden som gjorts för att komma fram till hur många deltagare som behövs för att uppnå tillräcklig statistisk styrka. Redogörelsen av hur man kommit fram till lämpligt urval ska beskriva projektets möjligheter att besvara frågeställningarna.

Då det inte finns några studier på sömnroboten än är det svårt att beräkna power. Resultaten från Delstudie 1 kommer vara vägledande för om vi behöver anpassa interventionen i Delstudie 2 och 3 (exempelvis kortare interventionsperiod). På grund av det låga antalet (n=4) kommer vi inte att kunna publicera resultaten från studien, men möjligen presentera de på en konferens.

Vad gäller Delstudie 2 planerar vi för 32 deltagare, som först fungerar som väntelistekontroller innan de får interventionen. Detta gäller alla deltagare förutom de första fyra som får interventionen, som inte kan vara kontroller. Eftersom de sista fyra som får interventionen inte har en parallell kontrollgrupp blir det fyra färre i den totala kontrollgruppen jämfört med interventionsgruppen (28 respektive 32). Forskargruppen kommer att rekrytera fler deltagare till deslstudie 2 om vi inte hittar en effekt av sömnroboten men inte heller kan utesluta att det finns en betydande effekt.

Till Delstudie 3 planerar vi att rekrytera 20 deltagare. Detta kommer vara en inomgruppsstudie med ett AB-design och då anses 20 deltagare vara tillräckligt för att uppnå statistisk styrka.

6.3. Hur kommer undersökningsprocedurerna att dokumenteras?

Redogör för hur undersökningarna och eventuella ingrepp dokumenteras. Ange om band- och videoinspelningar kommer användas.

De individuella intervjuerna efter interventionen i Delstudie 1 och 3 spelas in med diktafon. Svar på frågor från de strukturerade diagnostiska intervjuerna dokumenteras med papper och penna på de tillhörande formulären.

6.4. Hur kommer insamlad data att hanteras och förvaras?

Redogör för hur data kommer att hanteras efter insamlingen. Om data kommer att pseudonymiseras ("kodas") ska kodningsförfarandet beskrivas. Det ska framgå var kodlistorna/kodnycklarna kommer att förvaras samt vem/vilka som kommer ha tillgång till dem. Det ska även framgå hur länge data kommer att sparas samt om det kommer att avidentifieras (genom att kodnyckel förstörs) eller förstöras i sin helhet.

Enkäterna besvaras i plattformen Iterapi som har använts i andra forskningsstudier, och som bedöms vara en säker plattform (Vlaescu et al., 2016). Data kommer att behandlas och lagras i enlighet med dataskyddsförordningen. Data pseudonymiseras och kodnyckel med identifierande personuppgifter (vilken kod som hör till vilka personuppgifter) förvaras separat från datan. Ingen obehörig har tillgång till materialet. I enlighet med arkivlagen kommer data att sparas i minst 10 år efter studiens avslut och därefter förstöras.

7. Etiska överväganden

7.1. Vilka risker kan ett deltagande medföra för de forskningspersoner som ingår i forskningsprojektet?

Risker kan vara av olika karaktär. Det kan exempelvis vara fysisk eller psykisk skada, smärta, obehag, integritetsintrång på kort eller lång sikt. Ange vilka risker det kan finnas för de forskningspersoner som deltar i detta forskningsprojekt.

Ett forskningsetiskt övervägande gäller den enskilde individens upplevelse av integritetsintrång när känsliga frågor om personuppgifter, psykiska symptom och mående ställs. De formulär som används har använts i flera studier utan att problem upplevts på grund av frågornas karaktär. De individuella explorativa intervjuerna kommer att spelas in (ljudinspelning). Vår erfarenhet är att varken ljudinspelning eller intervjuer generellt upplevs som obehagligt. Projektet kommer att efterleva informationskravet, samtyckeskravet, konfidentialitetskravet samt nyttjandekravet.

Forskningsmaterialet (skattningsformulär, ljudinspelning och statistik från aktivitetsarmbanden) förvaras på två olika ändamålsenligt krypterade USB-minnen som är inlåsta i skåp som bara projektledaren har tillgång till. En sifferkod och kodnyckel med information om namn och personnummer upprättas för var av deltagarna. Kodnyckeln förvaras i ett låst arkivskåp i ett annat rum. Det är enbart forskare som arbetar med studien som kommer att kunna få ta del av resultaten för enskilda deltagare. Resultat redovisas både på gruppnivå och på individnivå (kvantitativa och kvalitativa data). I studier där resultaten presenteras på individnivå (till exempel citat vid intervjuer) finns risk att deltagare blir igenkänningsbara. Vi kommer därför att avidentifiera data och vara försiktiga med att förknippa enskilda resultat med specifika citat som skulle kunna göra att deltagarnas anonymitet röjs. Sammanställningen sker därmed på ett sätt så att enskilda individer inte kan identifieras.

Ett etiskt dilemma av klinisk relevans framförallt vad gäller Delstudie 3 är att studien erbjuder en behandling som deltagarna inte får fortsätta med efter studien även om sömnroboten skulle visa sig ha positiv effekt på patienternas symtombörda. Deltagarna kommer däremot efter studien att få information om sedvanlig behandling vid neuropsykiatriska svårigheter och insomni och råd om hur de kan gå tillväga för att få sådan hjälp. Det är också teoretiskt möjligt att sömnroboten har effekt även på längre sikt efter att man slutat använda den, för att man till exempel har lärt sig strategier för att slappna av eller komma ner i varv.

Vissa deltagare kan komma att bli besvikna om interventionen med sömnroboten inte ger förväntad effekt. För att minimera denna risk kommer forskningsgruppen att informera alla deltagare om detta möjliga utfall. Vissa deltagare kan också komma att förvärras under studiens gång antingen vad gäller deras sömnsymtom eller andra psykiatriska tillstånd. Forskningsgruppen består av tre psykologer, inkluderat en specialistpsykolog, som är vana att hantera sådana situationer och som kommer att hjälpa deltagarna att hantera sina känslor och symtom i stunden samt hänvisa deltagarna till rätt instans inom hälsa- och sjukvården om nödvändigt. Forskningsgruppen bedömer risken för en försämring på grund av sömnroboten att vara väldigt liten.

Personer som är intresserade av att vara med i studien men som uppfyller något eller några av exklusionskriterierna och därför inte får vara med kan uppfatta detta som negativt. Det finns en risk att de som exkluderas från studien inte söker annan mer passande hjälp. Alla som blir exkluderade kommer därför att bli aktivt guidade till var de kan söka mer adekvat hjälp.

Vad gäller smittsäkerhet har sömnroboten ett överdrag som tvättas mellan olika deltagares användning av roboten. Screeningen sker i huvudsak via telefon, alternativt i ett särskilt rum på Universitet med plexiglas mellan forskaren och deltagaren vid särskilt önskemål om ett fysiskt möte. Det finns tillgång till handtvätt och handsprit vid fysiska möten som i utgångspunkten endast sker vid hämtning och lämning av sömnroboten. Deltagare som har symtom på covid-19 uppmanas att informera forskargruppen snarast. I de fall då sömnroboten befinner sig hos någon sådan deltagare kommer gruppen att rådgöra med säkerhetschefen på KAU, angående lämplig hantering av sömnroboten. En sömnrobot som varit hos deltagare med symtom kommer inte lämnas ut igen förrän expert anger att så skulle vara säkert utifrån smittsynpunkt.

7.2. Vilken nytta kan ett deltagande medföra för de forskningspersoner som ingår i forskningsprojektet?

Ange vilken hjälp de forskningspersoner som deltar i detta forskningsprojekt kan få som ett resultat av forskningsprojektet.

Avslappning har generellt sett en välgörande effekt på hälsan, fysisk som psykisk. Både insomni och ADHD är förknippade med för hög uppvarvningsnivå. Att lugna ned sin andningstakt kan vara ett sätt att lugna ned sig och underlätta insomningen men det kan vara svårt att få till på egen hand, utan stöd. I projektet kommer deltagarna att få ta del av forskning som ligger i framkant inom insomniområdet och som kan förbättra vården för människor med sömnsvårigheter generellt, men kanske speciellt för personer med neuropsykiatriska svårigheter och samsjuklig sömnstörning som utgör en negligerad grupp inom sömnforskningen. Det är också möjligt att sömnroboten visar sig ha långsiktiga positiva effekter på deltagarnas sömn efter kortsiktig användning genom att deltagarna till exempel har lärt sig strategier för att varva ned.

7.3. Gör en värdering av förhållandet mellan riskerna och nyttan av projektet.

Forskning får enligt etikprövningslagen bara godkännas om riskerna som den kan medföra för forskningspersonernas hälsa, säkerhet och personliga integritet uppvägs av dess vetenskapliga värde.

Alla forskningsstudier kräver viss tid och innebär vissa risker för deltagarna. Tidsramen för deltagarna i vårt projekt är relativt kort, och de möjliga fördelerna med studien bedöms väga tyngre än de möjliga riskerna.

7.4. Beskriv hur projektet har utformats för att minimera riskerna för forskningspersonerna.

Forskning får enligt etikprövningslagen bara godkännas om det förväntade resultatet inte kan nås på ett annat sätt som innebär mindre risker för forskningspersoners hälsa, säkerhet och personliga integritet.

Behandling av personuppgifter som avses i 3 § får godkännas bara om den är nödvändig för att forskningen ska kunna utföras.

Observera att om en behandling ska prövas för första gången på människa måste detta tydligt framgå och relevanta säkerhetsrutiner tydligt beskrivas.

Ange vilka risker som ett deltagande i forskningsprojektet kan innebära. Finns det risker t.ex. med att ingå i en kontrollgrupp?

Forskargruppen undersöker successivt effekterna och användningssätten på ett fåtal personer under väldigt kontrollerade former i Delstudie 1, till att inkludera fler och ha bättre underbyggda instruktioner för deltagarna i Delstudie 2, till att slutligen i Delstudie 3 inkludera personer som kan anses mer sårbara och undersöker dessa mer noga. Alla deltagarna i alla tre delstudier kommer att få interventionen.

Forskargruppen består av tre psykologer (inkluderat en specialistpsykolog). Deltagarna uppmanas att kontakta oss vid frågor eller försämrat mående, och i sådana fall har forskargruppen beredskap att hantera situationen för stunden samt för att lotsa deltagarna vidare till adekvat hjälp.

7.5. Identifiera och precisera om eventuella etiska problem (nackdelar/fördelar) kan uppstå i ett vidare perspektiv genom forskningsprojektet.

Redovisa t.ex. om vissa grupper (andra än de forskningspersoner som ingår i forskningsprojektet) kan komma att utpekas respektive få hjälp som ett resultat av projektet. Frågan avser även de indirekta riskerna eller den indirekta nyttan. T.ex. genetisk påverkan på kommande generationer eller om resultaten på annat sätt kan tänkas skada vissa grupper.

Skulle sömnroboten visa sig ha god effekt på sömnen för deltagarna i vårt projekt kan det komma att utgöra ett behandlingsalternativ inom hälso- och sjukvården för bland annat personer med neuropsykiatriska svårigheter och samsjuklig sömnstörning som kanske inte kan eller önskar genomgå en KBT-I-behandling, och där befintliga läkemedel och medicintekniska produkter inte fungerar eller inte kan testas på grund av medicinska skäl. Resultaten från vårt projekt kan också komma att bli vägledande för framtida studier på sömnrobotar med andra typer av sömnsvårigheter och (neuro)psykiatriska tillstånd.

8. Forskningspersoner

8.1. Hur görs urvalet av forskningspersoner?

Forskningsperson: De levande människor som forskningen avser.

Beskriv vilka forskningspersoner som kommer inkluderas i projektet. Redogör för de överväganden som gjorts vid valet av forskningspersoner. Om vissa grupper utesluts från deltagande i projektet ska en motivering till uteslutandet framgå.

Redogör även för hur forskaren kommer i kontakt med eller får kännedom om lämpliga forskningspersoner.

Forskargruppen planerar att rekrytera fyra vuxna personer med insomni till Delstudie 1 och ytterligare 32 personer med insomni till Delstudie 2. Rekrytering kommer ske via sociala medier och annonser på campus på Karlstads universitet. Till de två första delstudierna önskar vi rekrytera relativt friska deltagare för att kunna utesluta andra komplicerande faktorers inverkan på resultaten. Olika mediciner och fysisk samt psykisk ohälsa har visat sig påverka sömnproblem och ibland kräva specialiserade insatser. Forskargruppen planerar att rekrytera 20 personer med ADHD och insomni till Delstudie 3. Rekryteringen kommer då ske via sociala medier och intresseorganisationer.

8.2. Hur många forskningspersoner kommer att inkluderas i forskningsprojektet?

Ange hur många forskningspersoner som totalt kommer inkluderas i projektet samt i förekommande fall hur många som kommer inkluderas i olika delprojekt.

4 försökspersoner i Delstudie 1, 32 försökspersoner i Delstudie 2, samt 20 försökspersoner i Delstudie 3.

8.3. Vilka urvalskriterier kommer att användas för inklusion?

Ange vilka kriterier som måste uppfyllas för att en forskningsperson ska inkluderas i projektet.

Personer som är intresserade av att delta i forskningsstudien kommer först att få genomgå en kortare screening, steg 1, som kommer bestå av självskattningsformulären Insomnia Severity Index (ISI) och Pre-Sleep Arousal Scale (PSAS). De personer som har förhöjda nivåer (över 8 på ISI och över 10 på den somatiska skalan i PSAS) kommer att kontaktas för att genomgå två strukturerade intervjuer via telefon:
- The Duke Structured Interview for Sleeping Disorders (DSISD), detta för att kontrollera att personer som inkluderas i studien har en diagnos av insomni, steg 2 men inte insomnisymtom som bättre kan förklaras av annan obehandlad sömnstörning.
- Mini International Neuropsychiatric Interview (M.I.N.I.), detta för att utesluta att deltagaren uppfyller några av exklusionskriterierna.

8.4. Vilka urvalskriterier kommer att användas för exklusion?

Ange vilka kriterier som måste uppfyllas för att en forskningsperson ska exkluderas ur projektet.

I de två första delstudierna utgör alla psykatriska och neuropsykiatrika diagnoser förutom insomni exklusionskriterier. Delstudie 3 har följande exklusionskriterier: pågående alkohol-, drog- eller läkemedelsmissbruk under de senaste tre månaderna, psykos, svår depression eller en aktuell bipolär symtombild samt suicidalitet. En eventuell pågående läkemedelsbehandling för ADHD och/eller sömnsvårigheterna ska vara välinställd (optimal dos under minst två månader).

8.5. Ange relationen mellan forskare och forskningspersonerna.

Ange forskarens roll i relation till forskningspersonens roll. Det kan t.ex. vara som behandlare (läkare, psykolog, fysioterapeut etc.) och patient/klient, som lärare och student eller arbetsgivare och anställd. All form av relation som kan tänkas medföra risk för påverkan ska beskrivas. Enligt etikprövningslagen ska information och samtycke ägnas särskild uppmärksamhet om forskningspersonen står i ett beroendeförhållande till huvudmannen, forskaren eller antas ha svårigheter att ta tillvara sin rätt.

Eftersom rekrytering till de två första delstudierna delvis sker på campus kan det visa sig att vissa deltagare är (tidigare) studenter av någon i forskningsgruppen. Ingen annan relation mellan forskare och forskningspersonerna.

8.6. Vilket försäkringsskydd finns för de forskningspersoner som deltar i forskningsprojektet?

Forskningshuvudmannen har ansvar för att kontrollera att det finns försäkring som täcker eventuella skador som kan uppkomma i samband med forskningen. Ange vilka försäkringar som forskningspersonen kommer att omfattas av samt vilket skydd försäkringarna ger forskningspersonen.

Patientskadeförsäkringen gäller.

8.7. Redogör för den beredskap som finns för att hantera oväntade bifynd eller händelser under forskningsprocessen som kan äventyra forskningspersonernas säkerhet.

Beskriv vilken tillgång forskningsprojektet har till utrustning, personal och kompetens för att hantera eventuellt oväntade komplikationer eller bifynd. Beskriv även vilken planering som finns för att hantera eventuella oväntade komplikationer eller bifynd.

Forskningsgruppen består av tre psykologer, inkluderat en specialistpsykolog, som är vana att hantera oväntade situationer både kliniskt och i behandlingsstudier.

I nuläget ligger eventuellt insjuknande i covid-19 som en påtaglig oväntad händelse som kan innebära åtgärder i projektet. Deltagare som har symtom på covid-19 uppmanas att informera forskargruppen snarast. I de fall då sömnroboten befinner sig hos någon sådan deltagare kommer gruppen att rådgöra med säkerhetschefen på KAU, angående lämplig hantering av sömnroboten. En sömnrobot som varit hos deltagare med symtom kommer inte lämnas ut igen förrän expert anger att så skulle vara säkert utifrån smittsynpunkt.

8.8. Kommer ekonomisk ersättning eller andra förmåner betalas ut till forskningspersonerna?

Forskningspersonerna kan utöver ersättning för resor, förlorad arbetsinkomst eller andra utgifter erhålla viss ersättning för obehag och besvär. Ersättningen ska vara skälig. Om barn eller ungdomar under 18 år deltar i forskningsprojektet får sådan ersättning inte vara stor och den bör inte erbjudas i samband med rekryteringen. Vid klinisk läkemedelsprövning med barn eller ungdomar under 18 år får inga incitament eller ekonomiska förmåner ges, undantaget kostnadsersättningar.

Nej

8.8.1. [Om Ja 8.8] **Vilken ekonomisk ersättning kommer betalas ut och när?**

Max 500 tecken

9. Information och samtycke

9.1. Kommer forskningspersonerna att informeras om forskningsprojektet och tillfrågas om de vill vara med eller inte?

Grundregeln, enligt etikprövningslagen, är att forskning bara utförs om forskningspersonen har informerats och samtyckt till deltagande. Information till forskningspersonerna kan ges både muntligt och skriftligt.

Ja

9.1.1. [Om Ja 9.1] **Hur, när (i vilket skede) och av vem informeras och tillfrågas forskningspersonerna?**

Beskriv proceduren för hur information ges och samtycke inhämtas. Vem som frågar, när detta sker och hur samtycket dokumenteras. På vilket sätt säkerställs att forskningspersonen ges betänketid och möjlighet att ställa frågor.

Utförlig redovisning är särskilt viktig när det ingår barn eller personer med nedsatt beslutskompetens i forskningsprojektet.

Skriftlig information om studien ges ut/hängs upp på campus vid Karlstads universitet och via sociala medier i de två första delstudierna, och i tillägg via brukarorganisationer i Delstudie 3. De som visar intresse för studien rings upp och får mer information om studien. Alla som är intresserade av att delta i studien får ytterligare skriftlig information om studien genom en websida. Forskningspersonerna kommer också att genomgå en längre telefonintervju där de får möjlighet att ställa frågor till intervjuande leg psykolog (eventuellt psykologstudent under handledning av leg psykolog) om studien. De får också betänketid gällande sitt deltagande innan de ger sitt skriftliga samtycke att delta i studien vid ett annat tillfälle.

9.1.2. [Om Nej 9.1] **Motivera varför forskningspersonerna inte ska informeras och tillfrågas.**

Lämna en utförlig redogörelse för de avvägningar som har gjorts och de skäl som ligger till grund för bedömningen att forskningspersonerna inte ska informeras och tillfrågas. Observera att forskning utan information och samtycke endast är möjlig i undantag fall och i den forskning sam avses i 3 § etikprövningslagen, eller 20–22 § i etikprövningslagen.

Max 4500 tecken

9.2. Kommer barn under 18 år att ingå i forskningsprojektet?

För barn under 15 år måste barnets samtliga vårdnadshavare ge sin tillåtelse (samtycka) till att barnet får delta i forskningsprojektet.

Barn mellan 15 och 18 år, som inser vad forskningen innebär för hans eller hennes del, ska informeras om och lämna eget samtycke till forskningen. I andra fall ska barnets samtliga vårdnadshavare ge sin tillåtelse (samtycka) till att barnet får delta i forskningsprojektet.

Forskningen får, trots vårdnadshavarnas tillåtelse (samtycke) inte utföras om barnet motsätter sig att forskningen utförs.

Nej

9.2.1. [Om Ja 9.2] **Ange barnens ålder.**

Max 500 tecken

9.3. Kommer forskningspersoner, vars mening på grund av sjukdom, psykisk störning, försvagat hälsotillstånd eller något annat liknande förhållande inte kan inhämtas, att ingå i forskningsprojektet?

Ange om forskningsprojektet kommer involvera personer som själva inte kan samtycka till sitt eget deltagande p.g.a. sjukdom, psykisk störning, försvagat hälsotillstånd eller liknande tillstånd.

Nej

9.3.1. [Om Ja 9.3] **Motivera varför denna grupp av forskningspersoner ska ingå i projektet.**

Forskning utan samtycke på denna grupp av forskningspersoner får endast utföras om forskningen förväntas ge en kunskap som inte är möjlig att få genom forskning med samtycke.

Forskningen ska dessutom förväntas leda till direkt nytta för forskningspersonen. Alternativt ska forskningen bidra till ett resultat som kan vara till nytta för forskningspersonen eller annan som lider av samma eller liknande sjukdom eller tillstånd samt innebära en obetydlig risk för skada eller obehag för forskningspersonen. Forskningspersonen ska så långt som möjligt informeras personligen om forskningen.

Samråd ska ske med närmaste anhörig. Samråd ska också ske med god man eller förvaltare om frågan ingår i uppdraget som god man eller förvaltare. Forskningen får inte utföras om forskningspersonen i någon form ger uttryck för att inte vilja delta eller om någon av dem som samråd har skett med motsätter sig införandet.

Max 2000 tecken

9.3.2. [Om Ja 9.3] **Beskriv hur samråd med närmaste anhörig, god man eller förvaltare kommer att ske.**

Vid forskning på forskningspersoner, vars mening inte går att inhämta, ska samråd ske med närmaste anhörig som ska få möjlighet att motsätta sig deltagandet. Samråd ska ske med god man eller förvaltare om frågan ingår i uppdraget som god man eller förvaltare.

Max 2000 tecken

10. Registeruppgifter

10.1. Kommer projektet att begära ut uppgifter från ett befintligt register?

Här avses alla typer av register som innehåller personuppgifter eller uppgifter som tidigare varit personuppgifter men senare avidentifierats.

Nej

10.1.1. [Om Ja 9.1] Ur vilket eller vilka register kommer uppgifterna att begäras?

Namnge registret eller registren som uppgifterna kommer att begäras ifrån. Ange även huvudman för respektive register.

Max 2000 tecken

10.1.2. [Om Ja 9.1] Vilka uppgifter kommer att begäras ut och varför?

Beskriv vilken typ av uppgifter som kommer att begäras ut och varför de behövs för att besvara projektets frågeställningar.

En komplett variabellista kan med fördel bifogas som bilaga. Etikprövningsmyndigheten behöver inte alltid en redovisning på variabelnivå för att göra sin bedömning, men det kan ibland underlätta vid begäran hos registerhållaren om den kompletta variabellistan funnits med vid etikprövningen.

Max 4500 tecken

11. Resultat från djurförsök

11.1. Finns det relevanta resultat från djurförsök?

Frågan avser framförallt klinisk behandlingsforskning. Om djurförsök inte har utförts, ange då anledningen till detta.

Ej Aktuellt

11.1.1. [Om Ja 11.1] Redogör för resultaten av djurförsöken

Redogör övergripande för de djurförsök som utförts och vilka resultat de gav. Framförallt ska de resultat som är av relevans för detta forskningsprojekt redogöras för.

Max 4500 tecken

12. REDOVISNING AV RESULTAT

12.1. Hur garanteras tillgång till data för forskningshuvudmannen och medverkande forskare?

Normalt ska den som ansvarar för genomförandet av forskningen ha full tillgång till data. Om flera forskare samverkar i uppdragsforskning bör den forskare som är huvudansvarig för genomförandet i förväg komma överens med övriga forskare om tillgång till data.

Alla forskarna i forskningsgruppen har full tillgång till data. Det är enbart forskare som arbetar med studien som kommer att kunna få ta del av resultaten för enskilda deltagare. Forskargruppen består av: Annika Norell-Clarke (docent i psykologi, leg psykolog), Maria Tillfors (professor i psykologi, leg psykolog), Erik Wästlund (docent i psykologi) och Siri Jakobsson Störe (doktorand i psykologi och specialistpsykolog).

12.2. Vem eller vilka ansvarar för databearbetning och skriftlig redovisning av resultaten?

Ange vem eller vilka som kommer bearbeta och analysera forskningsdata och vem eller vilka som kommer utforma den skriftliga redovisningen. Normalt ska den som ansvarar för genomförandet av forskningen ha full tillgång till data.

Huvudansvarig docent Annika Norell-Clarke, doktorand Siri Jakobsson Störe, professor Maria Tillfors och docent Erik Wästlund.

12.3. Hur och när planeras resultaten att offentliggöras?

Ange i vilken form resultaten planeras att offentliggöras. Exempelvis vetenskaplig publicering med peer review, open acess, intern rapport. Ange om möjligt en tidsplan för offentliggörandet.

Delstudie 1 planeras att redovisas verbalt på en vetenskaplig konferens tidigast hösten 2021. Resultaten från Delstudie 2 och 3 planeras att publiceras i internationella open access peer-review tidskrifter, hösten 2022-hösten 2023. Utöver detta kan resultaten komma att presenteras på nationella eller internationella konferenser.

12.4. På vilket sätt garanteras forskningspersonernas rätt till integritet när materialet offentliggörs?

Redogör för hur data presenteras när den offentliggörs och hur forskningspersonernas integritet skyddas vid offentliggörandet.

I studier där resultaten presenteras på individnivå (ex citat vid intervjuer) finns risk att deltagare blir igenkänningsbara. Forskningsgruppen kommer därför att avidentifiera data och vara försiktiga med att förknippa enskilda resultat med specifika citat som skulle kunna göra att deltagarnas anonymitet röjs. Vår gruppdesign medför att alla deltagare erbjuds interventionen. Sammanställningen sker därmed på ett sätt så att enskilda individer inte kan identifieras.

13. EKONOMISKA FÖRHÅLLANDEN

13.1. Redovisa eventuella ekonomiska överenskommelser med bidragsgivare eller andra finansiärer (namn och belopp).

Redovisa alla överenskommelser om finansiering som har slutits med den eller de som ska genomföra forskningen. Ange vilka belopp som kommer att erhållas för forskningsprojektet och vad ersättningen ska täcka. Ange även eventuella belopp per forskningsperson.

Ej aktuellt i nuläget.

13.2. Redovisa forskningshuvudmannens, huvudansvarig forskares och medverkande forskares egna ekonomiska intressen.

Redovisa egna ekonomiska intressen i form av t.ex. aktieinnehav, anställning, konsultuppdrag i finansierade företag, eget företag som kan få direkt eller indirekt vinst av forskningen.

Inga egna ekonomiska intressen.
