# Supplementary material for: A robot intervention for adults with ADHD and insomnia–A mixed-method proof-of-concept study
Source: PLoS One. 2023 Sep 1;18(9):e0290984. doi: 10.1371/journal.pone.0290984 (PMC10473504; doi:10.1371/journal.pone.0290984)
Supplement: S4 File — (DOCX) [file pone.0290984.s005.docx]

**Application for ethical review**

**Application for adjustment**

**1.1. Information about previous applications**

**1.1.1. Provide the reference number and decision date for the previously approved basic application.**

Reference number 2020-06975

Decision date 20/01/2021

**1.2. Parties**

**1.2.1. Specify the responsible researcher for the previously approved application.**

Annika Norell-Clarke

[annika.clarke@kau.se](mailto:annika.clarke@kau.se)

**1.2.3. Specify the research sponsor and authorized representative for the previously approved application.**

Karlstad University

Marie Nilsberth

Chair of the Research Ethics Committee

Associate Professor in Pedagogy

Marie.nilsberth@kau.se

**1.3. The title of the previously accepted application.**

Effects of the Somnox sleep robot on sleep and mental health in people with insomnia: with or without comorbid ADHD.

**1.4. Briefly describe the adjustments to the previously approved application.**

In the previously approved application, we described that participants in Study 1 would use actigraphs. We now wish to add that participants in Study 2 and 3 will also use actigraphs during parts of the studies.

Study 2…[Not translated]

**1.5. State the reasons underlying the planned adjustments.**

The changes can be considered significant amendments with regard to data collection.

**1.6. Evaluate how the balance between the risks and benefits of the project changes in light of the planned adjustments.**

We assess that the benefit of a more objective measure of sleep (by measuring movements during the night) is significant because subjective and objective measures of insomnia do not always correspond with each other. In addition, many treatment studies for people with insomnia use both objective and subjective measures. This allows us to compare our participants with other people with insomnia and thus gain more knowledge about how our group compares to others in terms of clinical level and treatment effectiveness.

There is a risk that some participants may find it uncomfortable to wear the actigraph and may not use it as intended. We will inform participants that they can stop wearing it at any time if it feels uncomfortable. We believe that most participants will get used to it.

Study 2…[Not translated]

**1.7. Describe, where applicable, how the information provided to research participants will change as a result of the planned amendment.**

The information provided to the research participants will only be changed by adding a sentence about actigraphs in the participant information.

Study 2…[Not translated]

**1.8. Describe, if applicable, how other information/attachments will be changed due to the planned amendment.**

We will also add a few sentences about actigraphs in the research plan.

**1.9. Signature and certification.**

**1.9.1. Specify the title of the previously approved application.**

Effects of the Somnox sleep robot on sleep and mental health in people with insomnia: with or without comorbid ADHD.

Signature from the responsible researcher.

Name: Annika Norell-Clarke

Date: 03/23/2021
