# Supplementary material for: A robot intervention for adults with ADHD and insomnia–A mixed-method proof-of-concept study
Source: PLoS One. 2023 Sep 1;18(9):e0290984. doi: 10.1371/journal.pone.0290984 (PMC10473504; doi:10.1371/journal.pone.0290984)
Supplement: S5 File — (DOCX) [file pone.0290984.s006.docx]

Ändringsansökan

1. Första steget när du ska ansöka om ändring är att fylla i [Ansökan om ändring](https://etikprovningsmyndigheten.se/wp-content/uploads/2019/09/andringsansokan-ansokan-om-etikprovning-av-andring.docx) (detta dokument).

Denna del av ansökan ska skrivas ut på papper för att sedan undertecknas av ansvarig forskare. Även behörig företrädare kan ansöka om ändring och då underteckna ansökan. Den undertecknade ansökan ska skannas in och göras sökbar, dvs. sparas som sökbar PDF-fil. Namnge ansökan ”Ändring” och projektets titel.

1. Nästa steg är att identifiera vilka bilagor som ska ändras. Dessa bilagor ska bifogas ansökan. Alla ändringar i bifogade dokument ska tydligt framgå, exempelvis genom funktionen spåra ändringar. Om bilagorna är omfattande ska det framgå på vilka sidor ändringarna är gjorda.

Samtliga bilagor ska sparas som sökbara PDF-filer. Om bilagorna är omfattande ska de även vara märkta med bokmärken (indexerade). Namnge alla bilagor med bilagenummer och namnet på bilagan, exempelvis ”02 beskrivning av forskningsprojektet”, ”03 forskningsplan”.

1. Den inskannade [Ansökan om ändring](https://etikprovningsmyndigheten.se/wp-content/uploads/2019/09/andringsansokan-ansokan-om-etikprovning-av-andring.docx) samt bilagor i sökbara PDF-filer skickas in i elektroniskt format till e-postadressen [ansokan@etikprovning.se](mailto:ansokan@etikprovning.se). Ge e-posten rubriken Ändringsansökan samt dnr på grundansökan.
2. Om ansökan rör en klinisk läkemedelsprövning kan du använda Eudralink när du skickar in ansökan. (för mer information om Eudralink se läkemedelsverkets webbplats för kliniska läkemedelsprövningar). Sätt förfallodatum så lång fram som möjligt och säkerställ att filerna inte är lösenordskyddade. Även dessa ansökningar ska skickas in till ansokan@etikprovning.se.
3. Myndigheten kan ta emot maximalt 50 000 KB (50 MB) i en e-post. Om din ansökan är större ber vi dig att försöka minska storleken eller dela upp ansökan i flera e-postmeddelanden. Ange då tydligt att e-postmeddelandena tillhör samma ansökan samt i hur många meddelanden som ansökan skickas in.
4. När Etikprövningsmyndigheten har tagit emot din ansökan kommer vi att skicka en bekräftelse via e-post till ansvarig forskare. I bekräftelsen får du information om vilket diarienummer din ansökan har fått samt hur du går tillväga för att betala avgiften för ansökan. Betalning ska alltid ske med angivande av OCR-nummer.
5. Först när avgiften är inbetald med korrekt OCR-nummer och finns på myndighetens konto kommer vi att påbörja handläggningen av din ansökan.
6. När vi har fattat ett beslut angående din ansökan kommer vi att skicka beslutet via e-post till ansvarig forskare.
7. Kom ihåg att ansökan alltid ska fyllas i på svenska och att ansökan ska vara förståelig för en lekman.

[**www.etikprovning.se**](http://www.etikprovningsmyndigheten.se/)

# Ansökan om etikprövning

Ansökan om ändring

- 1. Uppgifter om tidigare ansökningar
     1. Ange diarienummer och beslutsdatum på den tidigare godkända grundansökan.

Diarienummer 2020-06975

Beslutsdatum 2021-01-20

- - 1. Ange diarienummer och beslutsdatum för eventuella tidigare ändringsansökningar och ge en kort summering av vad de avsåg. Om antalet tidigare ändringar överstiger fem ska en separat förteckning över ändringsansökningarna biläggas.

- 1. Parter
     1. Ange ansvarig forskare för den tidigare godkända grundansökan.

Annika Norell-Clarke

annika.clarke@kau.se

- - 1. Om byte av ansvarig forskare har skett i en tidigare ändringsansökan: ange den nuvarande ansvariga forskaren.
    2. Ange forskningshuvudman och behörig företrädare för den tidigare godkända grundansökan.

Karlstads universitet

Marie Nilsberth

Ordförande i forskningsetiska kommittén

Docent i pedagogiskt arbete

marie.nilsberth@kau.se

- - 1. Om byte av forskningshuvudman har skett i en tidigare ändringsansökan: ange nuvarande huvudman och behörig företrädare.
    2. Om ansökan avser byte av ansvarig forskare: ange ny ansvarig forskare.
    3. Om ansökan avser byte av forskningshuvudman: ange namn och behörig företrädare hos ny forskningshuvudman.
  1. Ange titel på den tidigare godkända grundansökan.

Effekterna av somnox sömnrobot på sömn och psykisk (o)hälsa hos personer med insomni: med eller utan samsjuklig ADHD

- 1. Beskriv kortfattat den ändring av tidigare godkänd ansökan som planeras.

I den tidigare godkända ansökan beskriver vi att deltagarna i Delstudie 1 kommer att använda aktivitetsarmband (aktigrafer). Vi önskar nu lägga till att deltagarna i Delstudie 2 och 3 också kommer att använda aktigrafer under deler av studierna.

I tillägg önskar vi att deltagarna ska besvara några frågor om intag av koffein, alkohol och tobak (se bifogad fil) vid två tillfällen under studien, i samband med de övriga före- och eftermätningarna samt att frågeformuläret Insomnia Severity Index (7 frågor) besvaras vid en tidpunkt mitt under behandlingen för att kunna se hur sömnen förändras under behandlingens gång.

För att ta höjd för eventuellt bortfall så vill vi rekrytera ytterligare 12 personer till studien.

- 1. Ange de skäl som ligger till grund för den planerade ändringen.

Ändringarna kan anses vara väsentliga ändringar vad gäller insamling av data.

- 1. Gör en värdering av hur förhållandet mellan riskerna och nyttan av projektet förändras med anledning av den planerade ändringen.

Vi bedömer att nyttan med ett mer objektivt mått på sömn (genom att mäta rörelser under natten) är stor eftersom subjektiva och objektiva mått på insomni inte alltid motsvarar varann. I tillägg finns det många behandlingsstudier för personer med insomni som använder sig av både objektiva och subjektiva mått. Detta gör att vi kan jämföra våra deltagare med andra personer med insomni och därmed få mer kunskap kring hur vår grupp ligger realtivt andra vad gäller klinisk nivå och effekt av behandling.

Risken finns att vissa deltagare kan tycka att det är ovant att ha på sig armbandet, och kanske inte använder aktigrafen som det är tänkt. Vi kommer att informera deltagarna att de när som helst kan avbryta mätningen med aktigrafen om den upplevs som obehaglig. Vi tror att de flesta kommer att vänja sig.

Man får ut väldigt mycket data från aktigraferna, varför vi kommer skriva vilken data vi ska extrahera samt vilka hypoteser eller förväntade effekter vi har i en studieprotokoll när vi registrerar studien.

- 1. Beskriv i förekommande fall hur informationen till forskningspersonerna förändras med anledning av den planerade ändringen.

Informationen till forskningspersonerna ändras endast med att vi lägger till en mening om aktigraferna i forskningspersonsinformationen, samt en mening om ett extra mättillfälle mitt i behandlingen (Insomnia Severity Index, 7 frågor).

- 1. Beskriv i förekommande fall hur annan information/bilagor förändras med anledning av den planerade ändringen.

Vi lägger även till några meningar om aktigraferna i forskningsplanen.

- 1. Underskrift och intygande.
     1. Ange titel på den tidigare godkända grundansökan.

Effekterna av somnox sömnrobot på sömn och psykisk (o)hälsa hos personer med insomni: med eller utan samsjuklig ADHD

I och med att ansökan undertecknas intygar du som är ansvarig forskare följande:

Att den information som lämnas i ansökan om etikprövning och samtliga medföljande bilagor är riktig och fullständig.

Att verksamhetsansvariga i samtliga medverkande verksamheter är informerade om forskningsprojektets innehåll och utförande och att de har samtyckt till att delta i studien.

Att du säkerställt att det i samtliga medverkande verksamheter finns resurser som garanterar forskningspersonernas säkerhet och integritet vid genomförandet av den forskning som beskrivs i ansökan.

Att du tagit del av Etikprövningsmyndighetens information om hantering av personuppgifter på myndighetens webbplats.

–––––––––––––––––––––––––––––––––––––

Underskrift ansvarig forskare

Namn: Annika Norell-Clarke

Datum: 2021-03-23

–––––––––––––––––––––––––––––––––––––

Alternativt underskrift av behörig företrädare för forskningshuvudman

Namn: Namn Efternamn

Titel: Titel som innebär ett verksamhetsansvar

Datum: Datum

- - 1. Om ansökan avser byte av ansvarig forskare krävs ytterligare underskrifter och intygande.

Ange titel på den tidigare godkända grundansökan.

Om ändringen avser byte av ansvarig forskare ska ansökan även skrivas under av ny ansvarig forskare och behörig företrädare under denna punkt.

I och med att ansökan undertecknas intygar du som är ansvarig forskare följande:

Att den information som lämnas i ansökan om etikprövning och samtliga medföljande bilagor är riktig och fullständig. Att verksamhetsansvariga i samtliga medverkande verksamheter är informerade om forskningsprojektets innehåll och utförande och att de har samtyckt till att delta i studien. Att du säkerställt att det i samtliga medverkande verksamheter finns resurser som garanterar forskningspersonernas säkerhet och integritet vid genomförandet av den forskning som beskrivs i ansökan. Att du tagit del av Etikprövningsmyndighetens information om hantering av personuppgifter på myndighetens webbplats.

–––––––––––––––––––––––––––––––––––––

Underskrift ny ansvarig forskare

Namn: Namn Efternamn

Datum: Datum

–––––––––––––––––––––––––––––––––––––

Underskrift av behörig företrädare för forskningshuvudman

Namn: Namn Efternamn

Titel: Titel som innebär ett verksamhetsansvar

Datum: Datum

- - 1. Underskrift och intygande vid byte av forskningshuvudman.

Ange titel på den tidigare godkända grundansökan.

*Om ändringen avser byte av huvudman ska ansökan även* *skrivas under av behörig företrädare hos befintlig forskningshuvudman under denna punkt.*

*I och med att ansökan undertecknas intygar du som är behörig företrädare följande:*

*Att den information som lämnas i ansökan om etikprövning och samtliga medföljande bilagor är riktig och fullständig. Att verksamhetsansvariga i samtliga medverkande verksamheter är informerade om forskningsprojektets innehåll och utförande och att de har samtyckt till att delta i studien. Att du säkerställt att det i samtliga medverkande verksamheter finns resurser som garanterar forskningspersonernas säkerhet och integritet vid genomförandet av den forskning som beskrivs i ansökan. Att ansvarig forskare ges rätt att företräda huvudmannen i alla framtida kontakter med Etikprövningsmyndigheten som rör detta forskningsprojekt samt ansöka om ändringar i forskningsprojektet. Att du tagit del av Etikprövningsmyndighetens information om hantering av personuppgifter på myndighetens webbplats.*

–––––––––––––––––––––––––––––––––––––

Underskrift behörig företrädare för befintlig forskningshuvudman

Namn: Namn Efternamn

Titel: Titel som innebär ett verksamhetsansvar

Datum: Datum

- - 1. Underskrift och intygande vid byte av forskningshuvudman.

Ange titel på den tidigare godkända grundansökan.

Om ändringen avser byte av huvudman ska ansökan även skrivas under av behörig företrädare hos ny forskningshuvudman under denna punkt.

I och med att ansökan undertecknas intygar du som är behörig företrädare följande:

Att den information som lämnas i ansökan om etikprövning och samtliga medföljande bilagor är riktig och fullständig. Att verksamhetsansvariga i samtliga medverkande verksamheter är informerade om forskningsprojektets innehåll och utförande och att de har samtyckt till att delta i studien. Att du säkerställt att det i samtliga medverkande verksamheter finns resurser som garanterar forskningspersonernas säkerhet och integritet vid genomförandet av den forskning som beskrivs i ansökan. Att ansvarig forskare ges rätt att företräda huvudmannen i alla framtida kontakter med Etikprövningsmyndigheten som rör detta forskningsprojekt samt ansöka om ändringar i forskningsprojektet. Att du tagit del av Etikprövningsmyndighetens information om hantering av personuppgifter på myndighetens webbplats.

–––––––––––––––––––––––––––––––––––––

Underskrift behörig företrädare för ny forskningshuvudman

Namn: Namn Efternamn

Titel: Titel som innebär ett verksamhetsansvar

Datum: Datum
