# Supplementary material for: A robot intervention for adults with ADHD and insomnia–A mixed-method proof-of-concept study
Source: PLoS One. 2023 Sep 1;18(9):e0290984. doi: 10.1371/journal.pone.0290984 (PMC10473504; doi:10.1371/journal.pone.0290984)
Supplement: Attachment — Submitted filename: renamed_43c57.docx [file pone.0290984.s007.docx]

# COMMENTS – Manuscript PONE-D-23-08448

Title: “A robot intervention for adults with adhd and insomnia

– A mixed-method study”

**Important note: This review pertains only to ‘statistical aspects’ of the study and so ‘clinical aspects’ [like medical importance, relevance of the study, ‘clinical significance and implication(s)’ of the whole study, etc.] are to be evaluated [should be assessed] separately/independently. Further please note that any ‘statistical review’ is generally done under the assumption that (such) study specific methodological [as well as execution] issues are perfectly taken care of by the investigator(s). This review is not an exception to that and so does not cover clinical aspects {however, seldom comments are made only if those issues are intimately / scientifically related & intermingle with ‘statistical aspects’ of the study}. Agreed that ‘statistical methods’ are used as just tools here, however, they are vital part of methodology [and so should be given due importance]. I look at the manuscript in/with statistical view point, other reviewer(s) look(s) at it with different angle so that in totality the review is very comprehensive. However, there should be efforts from authors side to improve (may be by taking clues from reviewer’s comments). Therefore, please do not limit the revision only (with respect) to comments made here.**

**COMMENTS:** Although this manuscript is well drafted [and the study is excellent with respect to most of the aspects], I have few observations/concerns (different opinion) which are given below:

Firstly, I suggest a very minor correction. In the title [Title: “A robot intervention for adults with adhd and insomnia – A mixed-method study”] and subsequently at many more places [including at least five times in ‘Abstract’] ‘adhd’ is in small letters. It should be in capital letters ‘ADHD’ as it is a long-form/abbreviation which stands for ‘Attention Deficit & Hyperactivity Disorder’. Therefore, the required change is requested.

Since conclusion of this study [To conclude, the effects of the sleep robot intervention were mixed. More studies must be conducted on a modified version of the intervention before it can be recommended to adults with adhd and insomnia in clinical settings] is similar to those of quoted {both excellent and by the same team of scientists} studies (reference numbers 30 & 38 and thanks for giving DOI for both), I wish to know “why this study [though participant characteristics may be different] and moreover why on very small sample? Authors may have justification, but need to clarify.

Authors are likely to be aware of fact that: “Absence of evidence is not evidence of absence” [Altman DG, Bland JM. BMJ volume 311, 1995, p 485 (Reprinted: Australian Veterinary Journal 1996;74, 311)]. {Even when P-value is not significantly lower that is null hypothesis of no difference / no association is not rejected, (in short, result is not significant), that does not amount to evidence of absence i.e., it does not imply that there no difference / no association. It only implies that there is no (i.e., these samples do not provide) [say enough] evidence to prove (rather indicate with certain specified confidence level) the difference / association}. However, the is question is “HOW MUCH ETHICAL IT IS TO OFFER AN UNEFFECTIVE INTERVENTION TO WAIT-LISTED CONTROLS”? This question is not limited to your trial only and agreed that it may be the universal question. But how you dealt with ‘WAIT-LISTED CONTROLS’ in your trial is specific or pertaining to this trial and needs to be answered.

As you said “Considering the neurochemical and anatomical overlap in sleep, arousal and attention, it is possible that the robot might have greater effects in adults with comorbid adhd and insomnia” and I agree, however, the question of ‘small sample’ needs to be justified yet. You said “The sample size of 6 was determined based on the feasibility nature of the study” but then why title does/do not include/show that this is a feasibility study? It is also said that “The study was registered retrospectively due to the pilot nature of the study” but no mention of pilot nature of the study in ‘Abstract’. Considering variation in ‘Participant characteristics’ {ex. Age} and measurements (ex., The main outcome measure was WASO for Participant 1 and SOL for Participants 2-6) – tools or results/observations/recording - I sincerely of the opinion that it can be treated only as a pilot/feasibility study and needs clear mention in ‘Abstract’.

In ‘Intervention’ section it is stated that “Participants were free to use the robot for a shorter or a longer period of time”. Research methodology of ‘clinical trial’ can accept such point only (to some extent) if the trial is ‘pragmatic’. Except these minor points, the article is acceptable. However, mind you that as pointed out in ‘important note’ above “This review pertains only to ‘statistical aspects’ of the study and so ‘clinical aspects’ should be assessed separately/independently. ‘Minor Revision’ is recommended. Can be accepted only as a pilot/feasibility study after revision.
